# Enhancing Neoplasm Expression in Field Pea (*Pisum sativum*) via Intercropping and Its Significance to Pea Weevil (*Bruchus pisorum*) Management

**DOI:** 10.3389/fpls.2016.00654

**Published:** 2016-05-18

**Authors:** Abel Teshome, Tomas Bryngelsson, Esayas Mendesil, Salla Marttila, Mulatu Geleta

**Affiliations:** ^1^Department of Plant Breeding, Swedish University of Agricultural Sciences, AlnarpSweden; ^2^Department of Plant Protection Biology, Swedish University of Agricultural Sciences, AlnarpSweden

**Keywords:** *Bruchus pisorum*, field pea, intercropping, neoplasm, pea weevil, *Pisum sativum*

## Abstract

Neoplasm formation, a non-meristematic tissue growth on young field pea (*Pisum sativum* L.) pods is triggered in the absence of UV light and/or in response to oviposition by pea weevil (*Bruchus pisorum* L.). This trait is expressed in some genotypes [neoplastic (*Np*) genotypes] of *P*. *sativum* and has the capacity to obstruct pea weevil larval entry into developing seeds. In the present study, 26% of the tested accessions depicted the trait when grown under greenhouse conditions. However, UV light inhibits full expression of this trait and subsequently it is inconspicuous at the field level. In order to investigate UV light impact on the expression of neoplasm, particular *Np* genotypes were subjected to UV lamp light exposure in the greenhouse and sunlight at the field level. Under these different growing conditions, the highest mean percentage of *Np* pods was in the control chamber in the greenhouse (36%) whereas in single and double UV lamp chambers, the percentage dropped to 10 and 15%, respectively. Furthermore, when the same *Np* genotypes were grown in the field, the percentage of *Np* pods dropped significantly (7%). In order to enhance *Np* expression at the field level, intercropping of *Np* genotypes with sorghum was investigated. As result, the percentage of *Np* pods was threefold in intercropped *Np* genotypes as compared to those without intercropping. Therefore, intercropping *Np* genotypes with other crops such as sorghum and maize can facilitate neoplasm formation, which in turn can minimize the success rate of pea weevil larvae entry into developing seeds. Greenhouse artificial infestation experiments showed that pea weevil damage in *Np* genotypes is lower in comparison to wild type genotypes. Therefore, promoting *Np* formation under field conditions via intercropping can serve as part of an integrated pea weevil management strategy especially for small scale farming systems.

## Introduction

Abiotic factors like light, water and nutrients have a major influence on the phenotype of crops which in turn influences the multi-trophic interactions of the crop with ecological and economic implications (Dicke and Hilker, 2003). Some genotypes of field pea (*Pisum sativum* ssp. *sativum* L.) produce neoplasm on its pods when grown under greenhouse conditions. This phenomenon is a non-meristematic tissue growth on the surface of young pods in response to absence of UV light (Nuttall and Lyall, 1964; Dodds and Matthews, 1966). This trait can also be triggered by pea weevil (*Bruchus pisorum* L.) as a direct response to oviposition (Berdnikov et al., 1992; Doss et al., 2000). However, according to Doss et al. (1995), neoplastic (*Np*) tissue growth triggered by pea weevil oviposition is morphologically different from that caused by absence of UV light. Neoplasm in field pea is a result of mutation of a gene at *Np* (neoplasm) locus, in which a mutant allele (*Np*) is dominant over a wild type allele (*np*) (Nuttall and Lyall, 1964). Despite the fact that the expression of this trait is controlled by the dominant (*Np*) allele, its penetrance is influenced by the genotype (homozygosity: *Np*/*Np* vs. heterozygosity: *Np*/*np*), and the level of UV light intensity or humidity (Nuttall and Lyall, 1964; Burgess and Fleming, 1973; Doss et al., 2000). This trait has also been reported in other *Pisum* species like *P. elatius* and *P*. *humile* under greenhouse conditions (Dodds and Matthews, 1966).

Organic compounds extracted from pea weevil that were referred to as *bruchins* were reported to trigger neoplasm formation when applied on young pea pods of particular genotypes (Doss et al., 1995; Oliver et al., 2000; Doss, 2005). Doss (2005) reported that treating *Np* pea pods with *bruchins* lead to upregulation of genes that are known to be involved in defense metabolic pathways. In general, most studies conducted to date show that this trait is an induced response triggered by both abiotic and biotic stresses. Comparison of *Np* and wild type genotypes in their resistance to pea weevil revealed a lower average pea weevil damage in neoplasm producing genotypes under field (Doss et al., 2000) and greenhouse conditions (Teshome et al., 2015), which suggests the importance of this treat in pea weevil managment.

Pea weevil is the major menace in field pea production in Ethiopia and elsewhere (Pesho et al., 1977; Clement et al., 2002; Seyoum et al., 2012). Currently, chemical pesticide spraying and after harvest fumigation are the only options for pea weevil containment (Horne and Bailey, 1991; Baker, 1998). Despite the potential role of neoplasm formation in pea weevil management, little attention has been given to it until now. This is partly due to the fact that the penetrance of the trait under field conditions is inconspicuous and/or inconsistent (Nuttall and Lyall, 1964; Doss et al., 2000). In the present study, the influence of UV light on the expression of neoplasm under greenhouse conditions was studied in parental and F_1_ hybrids of *Np* and wild type (non-*Np*) genotypes. In addition, intercropping *Np* genotypes was investigated, if the shade provided by intercropping could enhance *Np* expression in *Np* genotypes under field conditions. Furthermore, both *Np* and wild type genotypes were screened for pea weevil resistance under greenhouse conditions.

## Materials and Methods

### Impact of UV Light on Neoplasm Formation

Neoplasm producing genotypes were identified from various field pea accessions during greenhouse screening experiments for pea weevil resistance in 2012 and 2013 (Teshome et al., 2015). The *Np* genotypes used in the present study are hereafter referred to as *Np* genotypes. The *Np* genotypes used in this study were 32433A, 203084A, 235899A, 237065A, 226037A, 226037B, 226037C, 226037D, and 226037E. Two separate experiments were carried out to study the effect of UV light on neoplasm formation. Primarily, *Np* genotypes were tested both under greenhouse and field conditions. Additionally, F_1_ hybrids produced from crosses of wild type genotype pollen recipient and *Np* pollen donor parents were tested for neoplasm formation under greenhouse conditions.

All plants were grown in 2 l plastic pots in a greenhouse chamber at 22°C and a minimum of 12 h light. Before flowering, plants were moved into chambers (2.54 m^2^) covered with a light-proof plastic sheet. Five chambers were used for this experiment with all having two cool white Fluorescent lamps, Sylvania Luxline plus 58W. One of the five chambers was used as a control chamber. The remaining four chambers had UV lamps, two of which had a single UV lamp (3 U 15 W UV light bulb) and the remaining two having double UV lamps (2 × 3 U 15 W UV light bulbs) each. The plants in the experimental chambers were exposed to UV light for 12 h from 6:00 pm to 6:00 am. Each genotype was represented by a minimum of three and a maximum of six replicates. Pods were harvested at maturity and individual pods of each genotype were assessed for neoplasm formation. Based on the level of neoplasm formation, pods from each plant were categorized into two different groups, low and high. The low score was given when there was sparse coverage of *Np* tissue on both sides of the pods. A high score was given when there was a conspicuous neoplasm formation that covered most of the pod. A similar protocol was used for the F_1_ hybrids as for the parental *Np* genotypes although they were only tested in double UV lamp and control chambers.

### Intercropping of *Np* Genotypes with Sorghum

Field experiments were carried out at Alnarp, Sweden in 2013 and 2014 to investigate the effect of shading provided by the canopy of sorghum on neoplasm expression. In this experiment, *Np* genotypes were intercropped with sorghum (*Sorghum bicolor* L. Moench.). Sorghum and pea genotypes were planted in different rows, in which each pea row had adjacent rows of sorghum on both sides. The distance between pea plants in a row was 5 cm and likewise in-between sorghum plants. The distance between rows was 10 cm. The total area of the plot was 2 m^2^ with two replications. Sorghum plants were also grown along the borders of the plots. There were five blocks in total with two blocks with intercropping and another two without intercropping and the last block with shading but without intercropping.

### Scanning Electron Microscopy (SEM)

Morphological and anatomical characteristics of *Np* pods, which were harvested at maturity from plants grown in the greenhouse, were examined under SEM. Small pieces of the pods from genotype 226037B were fixed with 2.5% glutaraldehyde in 0.1 M Na-phosphate buffer pH 7.2 overnight at +4°C, washed with the same buffer 3 × 15 min, dehydrated in graded series of ethanol and critical-point dried (CPD 020, Balzers, Lichtenstein). The samples were attached on the sample stubs with double-sided tape and sputter coated with gold and palladium 3:2 mix (JFC-1100, JOEL, Tokyo, Japan), and examined in a SEM (435 VP, LEO Electron Microscopy Ltd, Cambridge, UK) with 10 kV.

### Greenhouse Screening of *Np* and Wild Type Genotypes for Pea Weevil Resistance

Artificial infestation was done in insect rearing cages (60 cm × 60 cm × 120 cm, MegaView Science Co Ltd, Taiwan). The plants were moved into cages when they started to flower. Six plants were placed in each cage and each genotype was intermixed with different genotypes in consecutive experiments. Newly emerged adult pea weevils from seeds of a previous pea weevil screening study were used for artificial infestation. The weevils were kept at 4°C until they were released. In order to balance the sex ratio and ensure successful mating, the sex of the weevils was determined as described by Bousquet (1990) ahead of release. Twenty-five pairs of naive male and female pea weevils were released in each cage as soon as the first flower was detected. Pods were harvested at maturity and stored at room temperature. Three months after harvest, damage assessment was carried out on seeds of each genotype. Percent seed damage (PSD) was calculated based on pea weevil damage symptoms as described in Teshome et al. (2015). All plants used in this experiment were grown in a similar manner as in the UV light experiment.

### Data Analysis

All percentages of *Np* pods and PSD were arcsine transformed to homogenize variances and ensure normal distribution. A one way analysis of variance model was used to compare the proportion of neoplasm formation in *Np* genotypes when grown under different greenhouse and field conditions. In addition, pair-wise comparisons between the control, i.e., greenhouse normal lamp condition and all other conditions were carried out based on *post-hoc t*-test with *P*-values adjusted using the single-step method (Hothorn et al., 2008). A significance level of 5% was used for ANOVA and multiple comparison test. All analysis were carried on R version 3.2.2 (R Core Team, 2015).

## Results

**FIGURE 1:**
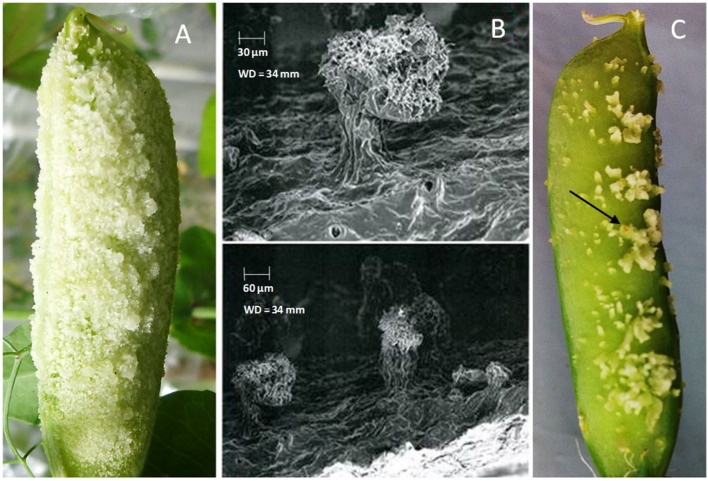
**(A)** Neoplasm formation in genotype 226037B pod in the control chamber (no UV light); **(B)** scanning electron micrographs of neoplasm formation on genotype 226037B; **(C)** pea weevil eggs oviposited on neoplastic (*Np*) pod in greenhouse screening.

Among 19 accessions used for resistance screening against pea weevil in the greenhouse, five accessions showed consistent neoplasm expression (**Table 1**). Neoplasm formation in these genotypes was clear and distinct (**Figure 1A**). The scanning electron micrographs also revealed a distinct outgrowth on the outer surface of pods of *Np* genotypes (**Figure 1B**). The remaining 15 field pea accessions as well as *P. fulvum* did not show any *Np* growth in repeated greenhouse experiments (**Table 1**).

**Table 1:** Comparison of average performance of neoplastic (*Np)* and non-*Np* genotypes originating from different accessions against pea weevil attack in greenhouse experiments.

Genotype	No. of plants	No. of seeds	Average PSD
32433^N^	4	137	12.0
235899^N^	3	194	12.5
226037^N^	36	1220	17.0
237065^N^	7	247	18.0
203084^N^	9	355	35.0
230846	3	22	4.5
32426	3	70	5.6
231277	4	158	6.0
230049	10	489	6.4
203083	3	257	6.7
32018	15	487	14.1
208459	2	130	15.0
32487	3	232	15.6
236413	7	274	17.1
32410	5	165	17.7
Adet	3	129	20.3
32063	14	286	20.7
2PW-2S-GR	7	229	31.8
32397	21	946	42.1
PF	4	42	8.3

*^*N*^refers to accessions with consistent *Np* formation, the rest are wild type (without *Np* formation). PF, *Pisum fulvum* genotype; PSD, percent seed damage.*

**FIGURE 2:**
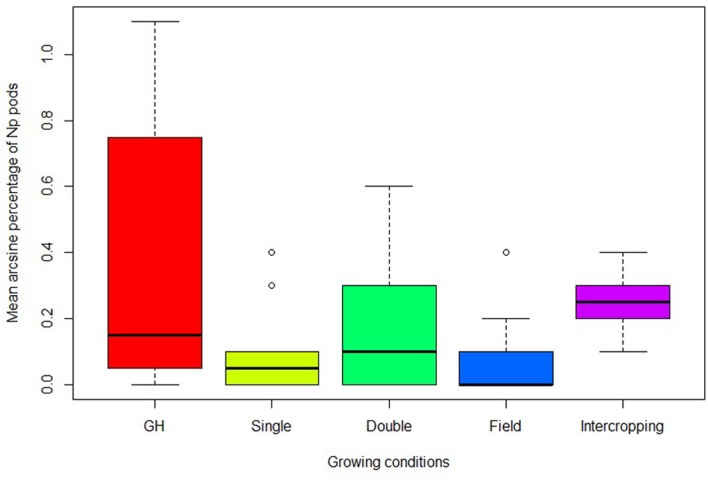
**Change in proportion of *Np* pods in different growing conditions in the greenhouse and at the field with and without intercropping.** Growing conditions; GH, control chamber; Single, single UV lamp; Double, double UV lamps; Field, field without intercropping; Intercropping, field with intercropping.

Particular *Np* genotypes that have shown consistent *Np* formation in repeated greenhouse trials were exposed to UV light to investigate UV influence on neoplasm formation. The highest percentage of *Np* pods, 36%, was recorded when genotypes were grown in the control chamber under greenhouse conditions and the least, 7%, when the replicates were grown in the field without intercropping. The mean percentage of *Np* pods in the field with intercropping was threefold of the mean *Np* pods without intercropping (**Table 2**). In addition, the median of the percentage of *Np* pods with intercropping was also higher than the median of the percentages recorded for the single and double UV light exposed chambers under greenhouse conditions (**Figure 2**).

**Table 2:** Pair-wise comparison of percent neoplasm formation on pods of selected genotypes grown under different greenhouse and field conditions with percent neoplasm formation on pods of same genotypes grown under normal light greenhouse condition (control).

Growing condition	Arc mean	Original mean	Standard error	*t*-value	*P*-value
Single UV lamp vs. Control	0.10	0.10	0.11	–2.5	0.05
Double UV lamps vs. Control	0.15	0.15	0.11	–2.1	0.14
Field without intercropping vs. Control	0.07	0.07	0.11	–2.8	0.03*
Field with intercropping vs. Control	0.25	0.25	0.11	–1.1	0.60

Arc mean, arcsine transformed mean for ANOVA. The arcsine and original mean of percent neoplasm formation of the control was 0.37 and 0.36, respectively. *Significantly different.

The comparison of the mean percentage of *Np* pods under greenhouse UV light exposure and field conditions revealed a marginally significant difference (*P* = 0.05). Further *post-hoc* test revealed that the mean percentage of *Np* pods without intercropping was significantly different from the control group in the greenhouse (**Table 2**). The *Np* genotypes grown under single or double UV lamps in the greenhouse or intercropped in the field were not significantly different from the control group in their mean percentage of *Np* pods. Under intercropping conditions, three genotypes 235899A, 237065A, and 22603B scored 30% or higher percent of *Np* pods. Interestingly, 203084A scored the highest percentage of *Np* pods (42.9%) in the field with intercropping but produced low percentage of *Np* pods in the control chamber (data not shown).

**FIGURE 3:**
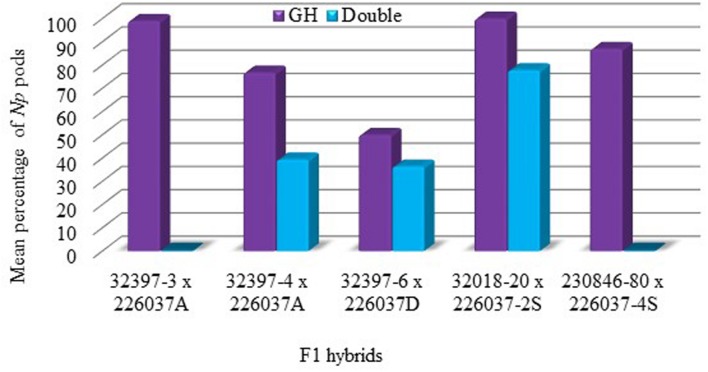
**Mean percentage of *Np* pods for F1 hybrids in the greenhouse.** Growing conditions; GH, control chamber; Double, double UV lamps.

All F1 hybrids produced by crossing non-*Np* (used as pollen recipient) and *Np* (used as pollen donors) produced neoplasm under greenhouse conditions (**Figure 3**). In both control and UV chambers, the highest percentage of *Np* pods was recorded for the F_1_ hybrid 32018-20 × 226037-2S with 100 and 77.8% *Np* pods, respectively. The least percentage of *Np* pods was scored for the 32397-6 × 226037D hybrid which was 50% in the control chamber. In most F_1_ hybrids, the percentage of *Np* pods decreased significantly when grown under double UV lamp conditions (**Figure 3**).

In general, *Np* accessions scored low average PSD in comparison to susceptible checks. The least average PSD among *Np* accessions was recorded for genotype 32433 which was 12% and the highest, 35%, for 203084 (**Table 1**). Among the wild type accessions, 231277, 32426, and 208459 recorded low PSD whereas 32397 (susceptible check) scored the highest average PSD (42%). After the initial screening that included both *Np* and wild type accessions, selected *Np* genotypes, a *P. fulvum* accession from NordGen and a *Np* F_1_ hybrid were tested for pea weevil resistance under greenhouse conditions. The *Np* genotypes scored less PSD in three consecutive greenhouse experiments with few notable exceptions (**Table 4**). The PSD of most *Np* genotypes was lower than the average seed damage all of plants in the same cage. For example, genotype 237065A scored 30% PSD at the first screening despite the mean PSD per cage and percent of plants with infested seeds per cage was 51 and 100%, respectively. Relatively lower PSD values were also observed for this genotype in the second and third round of screenings using seeds from the same generation. The *Np* F_1_ hybrid of 32397-3 × 226037A also scored a relatively low PSD in two consecutive experiments. On the contrary, 203084A, which is an *Np* genotype, scored high PSD values in all experiments. This genotype scored low percentage of *Np* pods in a control chamber in the greenhouse (**Table 3**). The *P. fulvum* genotype scored no seed damage in two consecutive experiments (**Table 4**). Analysis of variance (ANOVA) of PSD of *Np* genotypes, F_1_ hybrids and the *P. fulvum* line gave a highly significant variation, *P* = 0.001 (**Table 4**).

**Table 3:** Percentage of seed damage (PSD) of selected individual *Np* and wild type genotypes in three separate greenhouse experiments

Genotype	Screening	Neoplasm formation	PSD	MPSDC	PPISC
237065A	I	High	30	51	100
	II	High	46	54	83
	III	High	0	14.4	57.1
235899A	I	High	0	30	17
	II	High	37	31	100
	III	High	36.4	56.6	100
32433A	I	High	2	1	50
	II	High	9	11.2	66.7
	III	High	9	7	83
226037B	I	High	50	56.6	100
	II	High	45.5	72.2	100
	III	High	24	14.4	57.1
226037D	I	High	27	31	100
	II	High	0	2	33
	III	High	20	72	100
203084A	I	Low	93.3	72.2	100
	II	Low	74.5	57.5	100
	III	Low	73.3	75.5	100
32397^a^	I	No	80.5	73.6	100
	II	No	37	15.4	80
	III	No	27.8	26.9	75
PF	I	No	0	14.4	57.1
	II	No	0	26.9	75
32397-3 × 226037-3	I	High	1.5	20.6	66.7
	II	High	17.2	18.6	100

*^a^Non-*Np* genotype. PSD, percent seed damage; MPSDC, mean percent seed damage per cage; PPISC, percent of plants with infested seeds per cage.*

**Table 4:** ANOVA comparison of mean arcsine transformed PSD of genotypes tested in greenhouse screening.

	DF	Sum squares	Mean square	*F*-value	*P*-value
Genotypes	8	1.97	0.25	6.1	0.001**
Residuals	16	0.65	0.04		

**Highly significant.

## Discussion

Neoplasm formation is an infrequent phenomenon that occurs in certain genotypes of *P. sativum* in the absence of UV light, for example under greenhouse conditions (Nuttall and Lyall, 1964). **Figure 1A** shows *Np* tissue growth in *Np* genotype 226037B when grown under greenhouse conditions. In the present study, 26% of the tested *P. sativum* accessions depicted the trait (**Table 1**). According to Berdnikov et al. (1992), only 2.3% of the assessed Ethiopian germplasm collections showed neoplasm formation. The high percentage of *Np* accessions observed in the present study is most likely due to preselection of these accessions from a pool of collections used for resistance screening against pea weevil (Teshome et al., 2015).

The present study clearly showed that neoplasm formation on *Np* genotypes is conspicuous. However, the level of neoplasm varies among *Np* genotypes and growing conditions. A similar trend was reported in an oviposition preference study by Mendesil et al. (2016), where the level of neoplasm formation was different among *Np* genotypes. This study revealed that the highest proportion of pods with neoplasm was recorded when *Np* genotypes were grown in the control chamber under greenhouse conditions. In the control chamber, 36% of the pods showed neoplasm formation. On the contrary, when the same genotypes were exposed to single and double UV lamps, the proportion of *Np* pods was reduced. Furthermore, when these genotypes were grown under field conditions, the percentage of *Np* pods dropped to only 7%. This result is consistent with previous findings that reported a negative influence of UV light on neoplasm formation (Nuttall and Lyall, 1964; Dodds and Matthews, 1966).

Previous studies showed that the oviposition of female pea weevil on pods of *Np* genotypes triggers the expression of *Np* gene (Berdnikov et al., 1992; Hardie, 1992; Doss et al., 2000). However, the type of neoplasm formed on *Np* pods in the absence of UV light or upon oviposition by pea weevil is morphologically different (**Figures 1A,C**). Host plants usually trigger a series of responses to either prevent further oviposition or to reduce the success rate of deposited eggs (Hilker and Meiners, 2002; Fatouros et al., 2005; Meiners et al., 2005). According to Doss et al. (2000), neoplasm formation triggered by oviposition can impede the entry of newly hatched larvae and hence minimize infestation rate. Furthermore, the additional mass of cells on the epidermal layer of *Np* pods could potentially upset the behavior of the gravid female pea weevil when choosing site of oviposition. Oviposition preference experiments showed that *Np* genotypes had a reduced rate of oviposition as compared to wild type genotypes (Mendesil et al., 2016). Despite this trait being pertinent in pea weevil resistance, its expression is attenuated by UV light and hence less effective against pea weevil under field conditions (Nuttall and Lyall, 1964; Snoad and Matthews, 1969; Doss et al., 1995).

Nuttall and Lyall (1964) and Doss et al. (1995) detected neoplasm formation on shaded pods grown in the field. The present study has also showed an increase in neoplasm formation when the pods are shaded from direct sunlight at the field level (data not shown). However, mechanical shading is inconvenient for periodic application and resource and time consuming in the case of small-scale farming systems. On the other hand, the idea to shade the pods of *Np* genotypes with the canopy of taller and branching sorghum plants resulted in a significant increase in neoplasm formation. The proportion of *Np* pods with intercropping was three fold higher than without it at the field level (**Table 2**). The fact that neoplasm formation can be enhanced with intercropping, as shown in this study, indicates that intercropping can be implemented as part of an integrated pest management approach against pea weevil.

The fact that *Np* genotypes scored a relatively low PSD in three consecutive experiments under greenhouse conditions suggests intercropping as a viable approach in pea weevil management. Doss et al. (2000) reported that *Np* genotypes are less susceptible to pea weevil in comparison with wild type genotypes under field conditions. Similar results were also reported by Teshome et al. (2015) in an experiment conducted for screening field pea germplasm for resistance against pea weevil. Hence, enhancing *Np* formation with intercropping could be a way forward to minimize pea weevil damage at field level. Intercropping could also result in release of non-host volatiles that can adversely affect the pea weevil’s capability to locate its host and oviposit. According to Ali et al. (2007), intercropping field pea with different crops reduces susceptibility to *Ascochyta* blight and pea aphid (*Acyrthosiphon pisum*) infestation. Hence, intercropping is a silver bullet management option in field pea production that is environmentally benign, cost effective and requires minimum skill-set for application.

In order to successfully use neoplasm formation in field pea as part of integrated pea weevil management, the trait needs to be bred into locally adapted varieties. The experiment conducted to determine the heritability of neoplasm in field pea in the present study through crossing *Np* genotypes with wild type genotypes showed that all F_1_ hybrids produced neoplasm under greenhouse conditions suggesting that the *Np* allele at *Np* locus is dominant over wild type and the inheritance of the trait is according to the principle of Mendelian genetics, in line with previous studies (Nuttall and Lyall, 1964; Dodds and Matthews, 1966). However, similar to what was observed in the parental *Np* genotypes, the exposure of the F_1_ hybrids to double UV lamps results in a significant reduction in neoplasm formation (**Figure 3**), which signifies the importance of avoiding direct sunlight for effective expression of this trait under field conditions. The interspecific hybrids of *Np* field pea genotypes and *P. fulvum* also depicted neoplasm formation under greenhouse conditions. *P. fulvum* is known to have enhanced resistance against pea weevil as compared to cultivated *P. sativum* varieties (Hardie et al., 1995; Clement et al., 2002; Byrne et al., 2008). In the present study, both the *Np* genotypes and the *P. fulvum* line included in the artificial infestation experiment scored comparatively low PSD (**Table 4**). Therefore, developing field pea varieties through crossing *Np* genotypes with *P. fulvum* could result in pyramiding of resistance genes with different modes of action against pea weevil. Such varieties could have sustainable resistance and could easily be augmented by integrated pest management techniques like intercropping and trap cropping.

## Author Contributions

TB and MG secured the funding. AT and MG conceived and designed the study. AT, MG, EM, and SM collected the data. AT and MG performed data analysis. AT wrote the manuscript with help of MG, TB, EM, and SM.

## Conflict of Interest Statement

The authors declare that the research was conducted in the absence of any commercial or financial relationships that could be construed as a potential conflict of interest.
